# The SEARCH for Evidence: Designing a Game-Based Experiential Learning Intervention for Evidence-Based Medicine

**DOI:** 10.1007/s40670-026-02671-1

**Published:** 2026-03-05

**Authors:** Minhal Fatemah, Amal Fatemah, Saima Jawad

**Affiliations:** 1 Rawalpindi Medical University, Rawalpindi, Pakistan; 2 Lappeenranta-Lahti University of Technology (LUT), Lahti, Finland; 3 Bahria University, Islamabad, Pakistan

**Keywords:** Evidence-based medicine, Game-based learning, Experiential learning, Research literacy

## Abstract

Despite growing emphasis on research literacy in health professions education (HPE), learners often struggle with navigating scientific literature and applying evidence-based medicine (EBM) concepts. To address this gap, we developed the SEARCH framework (**S**elf-determined **E**xperiential **A**pproach for **R**esearch **C**ompetence in **H**ealthcare), grounded in experiential learning theory and self-determination theory (SDT). The SEARCH framework provides a structured, theory-informed approach to designing learning experiences to cultivate core research competencies.

To illustrate the framework’s application, we designed a virtual scavenger hunt as an exploratory game-based learning intervention to improve foundational research skills in undergraduate medical students and provide a hands-on exposure to EBM. Forty undergraduate medical students completed structured, interactive tasks targeting literature navigation, evidence appraisal and knowledge application. Participant task performance and reflective feedback were collected to demonstrate the feasibility of operationalizing the SEARCH framework in an authentic learning activity. Out of the forty participants, twenty-six completed the post activity survey. Participants reported high satisfaction and engagement. Thematic analysis revealed increased motivation, perceived skill development and engagement with the creative learning design. The task results indicated that while students performed well on knowledge-based tasks, challenges were noted in those requiring deeper article navigation and application of knowledge.

The SEARCH framework, therefore, offers a transferable model for integrating experiential, game-based approaches into research and EBM instruction.

## Introduction

In an ever-evolving medical landscape, medical professionals are tasked with making significant, life-saving decisions every day. Evidence-based medicine (EBM), defined as “conscientious, explicit and judicious use of current best evidence in making decisions about the care of individual patients” forms the cornerstone of medical decision making today [1]. Traditionally, the EBM cycle consists of 5 steps including *asking* a focused clinical question, *acquiring* evidence, *appraising* it critically, *applying* it and *assessing* outcomes [1, 2]. Without foundational skills in navigating and interpreting scientific literature, medical students may understand the *theory* of EBM but struggle to implement it in practice. This is the knowledge-application gap often observed in curricula [3, 4]. Research literacy therefore, remains crucial for equipping healthcare professionals with the skills to maintain an evidence-based approach when treating patients [5].

Traditionally, medical education emphasizes clinical knowledge and patient care and often does not apply the same evidence-based rigor to its own teaching practices [6, 7]. Moreover, despite its recognized importance and inclusion into undergraduate curricula, studies show that medical students have an average attitude towards EBM as reflected in their preferences for information sources during clinical decision-making and their perceptions of EBM practices [8]. Many medical students graduate with limited exposure to research methodologies and critical appraisal skills, resulting in inadequate preparation for conducting research and inability to integrate research findings into clinical decision making [9]. Given the increasing emphasis on EBM, this is particularly concerning.

To address persistent challenges in motivating medical students to meaningfully engage with EBM and research practices, we developed the SEARCH framework (**S**elf-determined **E**xperiential **A**pproach for **R**esearch **C**ompetence in **H**ealthcare). Grounded in Self-Determination Theory (SDT) [10] and Kolb’s Experiential Learning Theory (ELT) [11], the framework integrates motivational and pedagogical principles to provide hands-on exposure to and foster engagement with EBM.

Constructivist theories of education propose that knowledge is actively constructed through experience rather than passively received [12]. Social constructivism further highlights the importance of collaboration in learning, viewing knowledge as co-constructed through social interaction [13]. In EBM, this implies students should actively engage in tasks like formulating questions, analyzing data and interpreting results. ELT further supports this idea by advocating for *learning through doing* [11]. Studies have shown that experiential learning enhances long-term retention of knowledge and allows for deeper understanding of complex concepts, making it particularly suited for research literacy and EBM [14, 15].

To better understand what drives or hinders engagement in such contexts, we draw on Self-Determination Theory. SDT provides a framework for understanding how intrinsic motivation can be enhanced in educational settings, suggesting that students are more likely to engage deeply with learning materials when their needs for autonomy, competence and relatedness are met [10, 16]. Therefore, motivation is a key factor influencing student engagement with EBM since research is largely perceived as daunting, abstract and disconnected from clinical practice, leading to low interest and participation [9]. Within this motivational framework, Game-Based Learning (GBL) has gained attention as an innovative educational strategy [17, 18]. GBL is broadly defined as the use of game play with defined learning outcomes [19]. A core principle in GBL is balancing the instructional objectives with the immersive and engaging elements of gameplay [20]. By integrating features such as challenges, feedback, rewards, collaboration and competition, GBL has been shown to increase student engagement and motivation [21–23], teamwork [24], promote problem-solving abilities [25] and improve knowledge retention [26].

GBL strategies such as escape rooms have been garnering attention in medical educational settings [27–30]. These game-based strategies create an experiential learning environment that requires participants to navigate dynamic challenges and collaborate with peers to achieve a common goal. Various studies show the effective engagement of escape rooms as a tool to impart medical research education [29, 31, 32].

While escape rooms have been explored as game-based tools to teach clinical reasoning [33] and teamwork [28], scavenger hunts remain underutilized. Both approaches are goal-directed activities that offer opportunities for autonomous exploration, collaborative problem-solving and task-based challenges, which can support the psychological needs for autonomy, competence and relatedness. Escape rooms are typically characterized by a fixed space, linear or sequential task progression and time-limited completion, which may introduce cognitive pressure alongside learning objectives. In contrast, scavenger hunts emphasize exploratory, non-linear task completion, and flexible pacing, potentially allowing learners greater opportunity for reflection and self-directed engagement.

Although there is some evidence of the use of scavenger hunts as an educational tool in disciplines such as marketing [34], language learning [35] and computer science engineering [36], their use in the context of EBM education remains underexplored. For the purpose of this study, we define scavenger hunt as an interactive activity in which participants solve clues or complete tasks to locate specific information, objects or destinations.

Drawing on the proposed SEARCH framework, we designed and implemented a virtual scavenger hunt as a GBL intervention to promote core EBM and research literacy skills in undergraduate medical students in a developing country. While scavenger hunts can be implemented in both in-person or virtual formats, this study adopted a virtual approach. A virtual format was preferred in order to address logistical constraints associated with in-person delivery, including scheduling, equipment and space requirements. In addition, the online format offered increased flexibility and scalability, allowing participation across geographically distributed learners while supporting self-paced engagement.

This study introduces the SEARCH framework and illustrates its application in the design of a game-based educational intervention. It further reports on an exploratory evaluation to assess the interventions perceived educational impact and relevance in undergraduate medical research education.

## Methodology

This study employed a theory-driven, exploratory educational intervention to operationalize the presented SEARCH framework. The intervention was delivered as a short term, field-based learning activity conducted in a virtual, game-based format. The framework development and activity implementation are described below.

### Framework Development

The SEARCH framework was developed iteratively. An initial set of framework domains and design principles was derived from a review of the literature on game-based learning and instructional design in health professions education. Expert input was provided through consultation within the authorship team and with local medical educators. In particular, one author (S.J) is a senior educator with over three decades of experience in higher education including experience integrating game-based learning approaches across undergraduate curricula. This author has also contributed to the development and application of institutional frameworks for student-centered effective learning and internal quality culture in higher education [37, 38]. This expertise guided the articulation of framework components.

The framework was further refined by undergoing two rounds of pilot testing. The first pilot involved the design and implementation of an in-person scavenger hunt, followed by a second pilot consisting of a small-scale virtual scavenger hunt. These pilot applications were used to examine the clarity and usability of framework components for educators, and their alignment with intended EBM learning outcomes. Feedback from each pilot iteration was reviewed through discussion and manual concept mapping, leading to the final version of the SEARCH framework presented in this study.

### Scavenger Hunt Planning

#### Educational Setting

The activity was conducted with undergraduate medical students at Rawalpindi Medical University, Pakistan in June 2023, during summer break, to ensure maximum participation. It was conducted as part of a Virtual Research Olympiad conducted by the university’s student research society. The activity was promoted on the society’s social media platforms and students could register via an online Google form for free. It attracted participants from various national medical colleges, thereby providing a multi-institutional perspective. Participation was voluntary and the activity was conducted remotely in a fully virtual format.

#### Curriculum and Task Design

The instructional content of the virtual scavenger hunt was structured around the five core steps of EBM: Ask, Acquire, Appraise, Apply and Assess. Learning topics were selected to reflect key components of undergraduate medical research literacy, including formulation of clinical questions, literature searching, study design and statistical test identification, and data interpretation. The tasks were designed as riddle-based challenges requiring participants to locate, interpret and apply information from authentic research sources, including peer-reviewed journal articles and a provided dataset. Tasks were structured into layered subtasks to mirror Kolb’s experiential learning cycle, prompting active engagement, reflection, and re-application of concepts. This is discussed further in the 'Discussion' section.

Tasks aligned with the Ask and Acquire steps focused on formulating structured clinical questions using the PICO (Population, Intervention, Comparison and Outcome) framework, identifying appropriate MeSH terms, constructing database search strategies, and retrieving relevant literature from biomedical databases. The Acquire step was further reinforced through tasks requiring interpretation of bibliographic citations and extraction of key reference details.

The Appraise step constituted the largest proportion of activities and engaged participants in identifying appropriate statistical tests, recognizing study designs, evaluating data visualizations, and extracting reported outcomes from published research articles. These tasks required learners to interact directly with primary literature and apply methodological reasoning rather than rely on surface-level recognition.

Finally, participants engaged with the Apply step through a data analysis task which required them to conduct statistical testing, interpret findings, and make evidence-informed screening recommendations. Notably, the tasks were intentionally structured to require critical thinking, contextual understanding, engaging with published literature and data interpretation which are skills that could not be easily outsourced to AI tools.

The task set underwent review and refinement through one round of pilot application using a small-scale virtual scavenger hunt. This pilot was conducted to evaluate task clarity, feasibility, alignment with intended EBM learning objectives, and appropriateness of difficulty. Feedback from the pilot implementation informed revisions to task wording, sequencing, and instructional scaffolding prior to finalization of the task set.

**Fig. 1:**
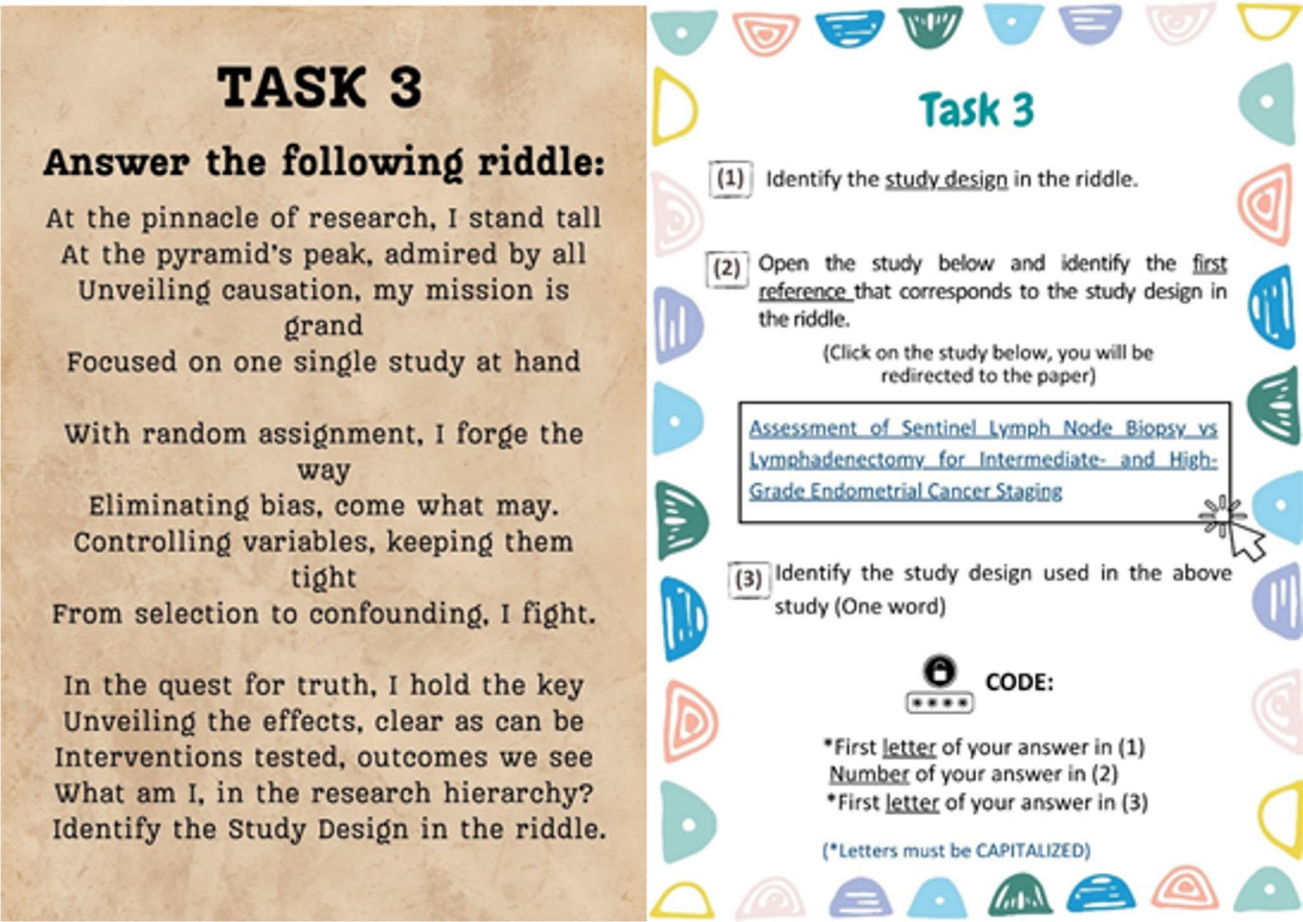
Example riddle and associated subtasks

Representative examples of tasks and their associated learning objectives are provided in Appendix (Table 2), while Fig. 1 illustrates an example task and its associated subtasks.

### Scavenger Hunt Implementation

#### Participants

A total of 40 undergraduate medical students participated. Participants ranged from second-year to final-year students, resulting in a heterogeneous cohort with varying levels of prior exposure to research and EBM. Participants worked in pairs, forming 20 teams that collaborated throughout the activity. Upon registration, students were given the option to self-select a partner; participants who did not select a partner were randomly paired by the organizers.

#### Implementation

Participants were first enrolled on an online platform that served as the communication hub for announcements and coordination. Prior to the event, participants received an introductory video outlining the objectives, rules and structure of the hunt. Supplementary instructional videos were provided to support scaffolding, and participants were permitted to consult any external online resources during task completion.

Throughout the activity, organizers provided real-time support through the communication platform, offering clarifications and hints as needed, to maintain engagement and ensure progress. Each participating pair was evaluated based on the accuracy and timeliness of their submissions. To foster a sense of achievement and encourage broad participation, all participants were awarded digital certificates of participation and top-performing participants were awarded shields.

**Fig. 2:**
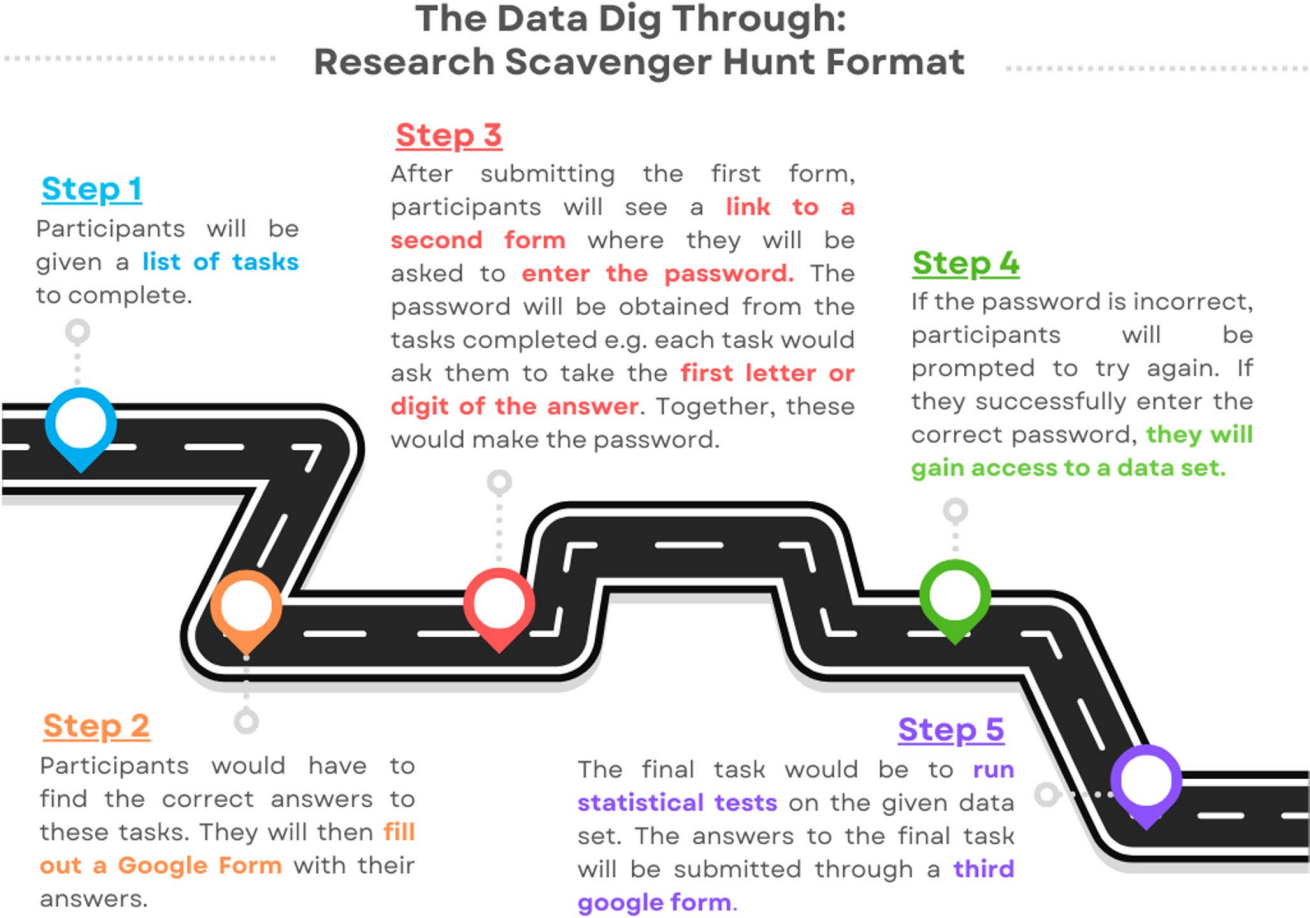
Blueprint of the virtual scavenger hunt

The working of the Virtual Scavenger Hunt is discussed in detail below and depicted in Fig. 2.

Step 1: Each pair of participants received a taskbook containing six riddles which were accompanied by subtasks. The subtasks within each riddle targeted specific research competencies, as mentioned in earlier in the 'Planning' section.

Step 2: Completion of these tasks, required participants to extract and analyze data from provided research articles and engage with shared instructional resources. Answers to these riddles and tasks were submitted through a Google form (Form 1).

Step 3: On submitting the answers, the participants were prompted to “enter the password” in a second Google form (Form 2). This password was an alphanumeric string comprising of “codes” that were obtained through solving the subtasks. Each correctly solved subtask contributed a specific alphanumeric character based on an accompanying rule (e.g., extracting a specific letter or number from the correct response). These characters collectively formed a password required to progress to the next phase of the scavenger hunt. For example, if the answer to a subtask was “Scavenger Hunt”, the accompanying rule might prompt the participant to take the first letter of the answer (i.e. the *code* would be the letter ‘S’). The final password would be the combination of the codes obtained from all of the tasks.

Step 4: Participants entered the assembled password into Form 2. If the password was entered correctly, participants were granted access to the subsequent phase of the activity, referred to as the “treasure chest,” which consisted of a shared drive containing a dataset and an additional taskbook. If the password was entered incorrectly, participants were notified immediately and prompted to review their previous responses and resubmit the password. No limit was placed on the number of password entry attempts.

Step 5: The second phase of the hunt, accessible only upon successful completion of the initial riddles, focused on data analysis and interpretation. Participants were required to apply statistical tests to the dataset, interpret their findings and answer a set of related questions. Responses were submitted through a final Google Form (Form 3). The participating pair who were the first to upload the correct answers were declared the winners.

### Data Acquisition and Analysis

Task performance data were collected from participants’ submissions across the three Google Forms used during the activity. These included accuracy of responses to riddle-based tasks, successful completion of subtasks, and correctness of the final data analysis outputs.

Perceptual data were collected using a post-activity survey administered after completion of the scavenger hunt. The survey comprised Likert-scale items (1 = Strongly Disagree to 5 = Strongly Agree) assessing participants’ satisfaction and perceived difficulty of the activity, alongside open-ended questions inviting reflection on learning experiences and perceived educational value.

Quantitative data from task performance and Likert-scale survey items were analyzed descriptively using summary statistics to characterize overall trends in performance and demonstrated competencies. Qualitative responses were analyzed using reflexive thematic analysis following Braun and Clarke’s approach [39, 40]. Coding was inductive and iterative, with reflexivity maintained throughout.

### Ethical Considerations

This study was conducted in accordance with the Declaration of Helsinki and the Finnish Advisory Board on Research Integrity (TENK) guidelines. Participation was voluntary and informed consent was obtained. Confidentiality was maintained and no harm or adverse consequences were associated with participation. Furthermore, the study did not affect academic assessment or grading. The activity was organized in collaboration with the university’s student research society, under supervision and approval of the university administration and faculty members. The study was assessed by local medical education researchers and in accordance with the national research ethics guidelines specific to such interventions in Pakistan, it was determined that the study constitutes minimal-risk educational research and was thus deemed exempt from full IRB review.

## Results

A total of 40 medical students participated in the virtual scavenger hunt, forming 20 participating pairs. Of these participants, 27 identified as female and 13 as male. The cohort included students from a range of academic years, with 30% (12) from second year, 12.5% (5) from third year, 42.5% (17) from fourth year and 15% (6) from final year.

### Task Performance

Performance was evaluated based on the accuracy of responses submitted. Table 1 summarizes the percentage of participating pairs that correctly answered each task. Common errors observed are also listed in the table.

**Table 1:** Summary of performance per task

Task No.	Skill Targeted	% Correct (No. of teams)	Common Errors
1	ANOVA	95% (19)	-
2a	PICO Table/ MeSH Term	65% (13)	Inability to differentiate between MeSH terms and Supplementary concepts
2b	Search string formulation	30% (6)	Did not understand how to apply Boolean operators
3a	Citation styles	60% (12)	Confusing similar formats
3b	Extracting bibliographic information	90% (18)	-
4	Data Visualization	85% (17)	-
5a	Study Design	70% (14)	Confusion between RCT and SRMA as top of research hierarchy (Riddle mentions *single study at hand*, See Fig. 1).
5b	Extract information/study design from bibliography	45% (9)	Inability to extract information from bibliography
5c	Identify study design of the given article	90% (18)	-
6a	Logistic Regression	90% (18)	-
6b	Odds Ratio	80% (16)	-
6c	Highest value of reported outcome in the given article	25% (5)	Skimming instead of analyzing
7	Data Analysis	15% (3)	-

### Participant Feedback

Of the 40 participants, 26 completed the post-activity survey (response rate: 65%). Responses offered insights into participant satisfaction, perceived difficulty and the perceived educational value of the scavenger hunt. The qualitative responses were thematically analyzed and organized into four categories: research skills, creative learning design, engagement & motivation.

#### Satisfaction and Perceived Difficulty

A majority of responses (85%, 22 out of 26) reported moderate to high satisfaction with the scavenger hunt. Perceived difficulty varied, with 69% (18 out of 26) rating the scavenger hunt as difficult or very difficult. 31% (8 out of 26) found it moderate to easy and none rated it as very easy.

#### Thematic Insights

##### Research Skills

Fourteen respondents (53.8%) described aspects of research skill development in their open-ended responses. Reported skills included problem-solving, critical thinking, persistence and collaboration. Several participants particularly appreciated the “*code for finding password*”, describing the experience as requiring “*brainstorming*” and perseverance, with one participant noting, “*We tried 100 out of 100 times in search of treasure!*”

##### Creative Learning Design

Approximately 35% of respondents commented on aspects of the scavenger hunt’s design in their open-ended responses. Responses emphasized its creative use of riddles, open-book structure and poetry-based clues. One participant commented, “*The idea of a scavenger hunt as an open-book quiz was brilliant*,” while another remarked, “*Boring analysis turned into a treasure hunt!*”. Several responses described the activity as an engaging *alternative assessment method*, offering a departure from traditional evaluation formats and promoting active learning.

##### Engagement & Motivation

Nearly one-third (31%) of respondents reflected on how the activity enhanced their motivation and enjoyment. Participants described it as “*thrilling*,” “*memorable*,” and “*a very unique event*,” with one noting, *“I loved everything about it and will definitely participate again in the future.”*

## Discussion

As the well-known proverb goes, “*I hear and I forget*,* I see and I remember*,* I do and I understand.*” This timeless insight highlights the value of experiential learning, yet, despite its growing adoption and the formal inclusion of EBM in medical curricula, a knowledge-application gap persists [8].

**Fig. 3:**
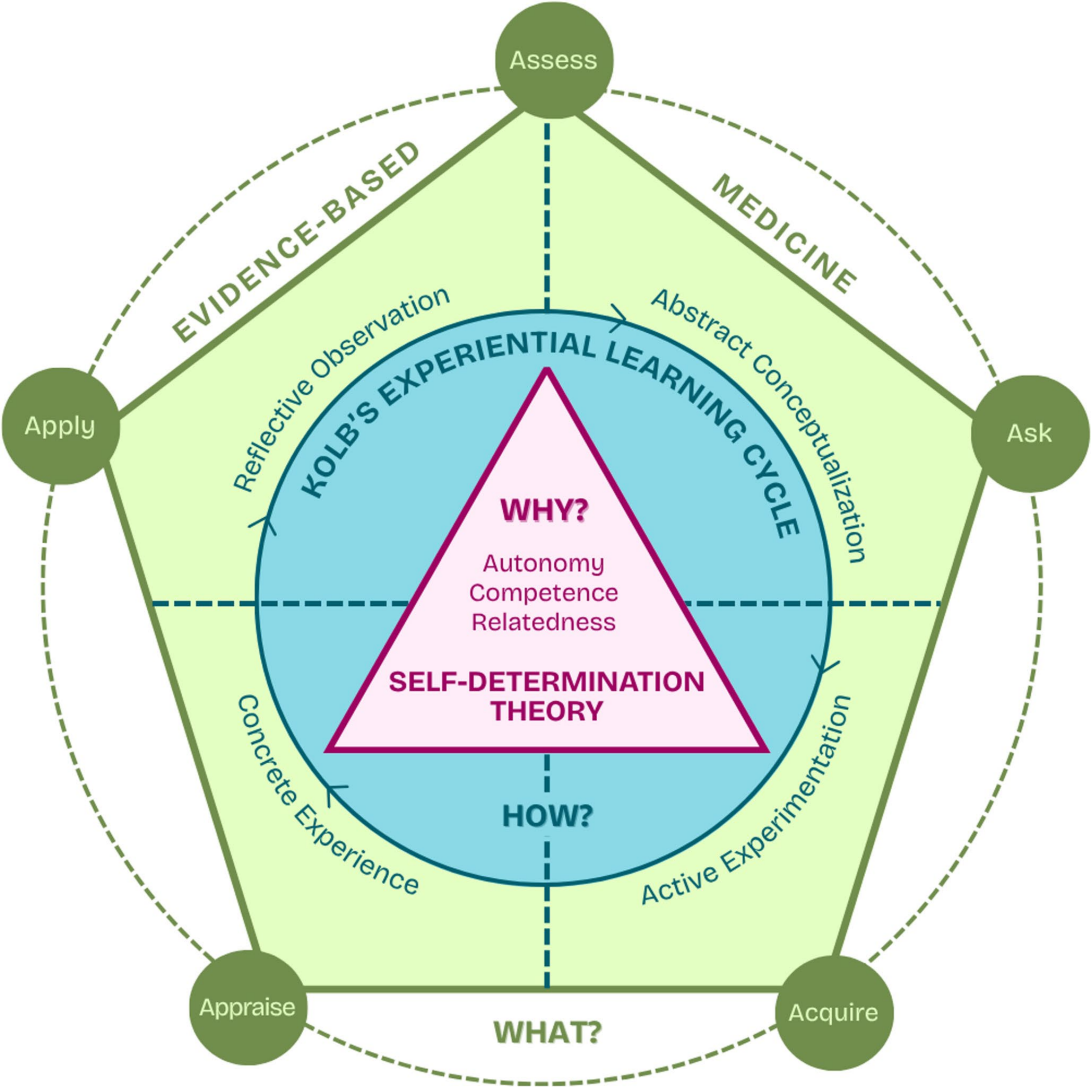
**S**elf-determined **E**xperiential **A**pproach for **R**esearch **C**ompetence in **H**ealthcare (SEARCH) framework

To address this gap, we conducted a virtual scavenger hunt designed to provide hands-on engagement with EBM to undergraduate medical students. To guide the design of the intervention, we developed a conceptual framework grounded in experiential learning theory and SDT [10] while utilizing GBL [17] and the five steps of EBM, thereby aligning motivation, pedagogy and content. This three tiered framework served both as a design scaffold and an interpretive lens, linking the “why” (SDT), “how” (experiential learning) and “what” (EBM competencies) of the activity. The proposed model is depicted in Fig. 3 and is discussed in detail below.

### SEARCH Framework

SDT forms the motivational core of this framework by addressing *why* learners engage with research tasks in the first place. As outlined in the introduction, students often approach research with low motivation, perceiving it as abstract and disconnected from clinical practice [9]. By designing tasks that fulfill psychological needs for autonomy, competence and relatedness, the activity aims to create a motivational climate that can encourage meaningful and sustained engagement [16]. Our choice of game mechanics is also supported by the GATE framework [41]. Minimal rules enabled learner *autonomy* since participants had the autonomy to navigate tasks independently, promoting a sense of ownership over their learning. The progressive challenges were designed to build *competence* by offering achievable difficulty, immediate feedback and instructional scaffolds. Collaboration and team-based problem-solving supported *relatedness*, encouraging social connection and shared goals.

Within the proposed SEARCH framework, Kolb’s Experiential Learning Theory informs *how* learning occurs. ELT conceptualizes learning as a cyclical process through which knowledge is constructed by transforming experience. The cycle comprises four interconnected stages: concrete experience, reflective observation, abstract conceptualization and active experimentation [11]. The virtual scavenger hunt was designed such that each major task was broken down into interdependent subtasks. These subtasks encouraged participants to engage actively, reflect on what they were doing or why a particular answer was incorrect (Concrete Experience and Reflective Observation), form understanding of underlying research principles (Abstract Conceptualization) and apply insights in a new but related challenge e.g. article navigation (Active Experimentation). A notable design feature was the use of password-protected “treasure chests,” that could only be unlocked by solving a series of interlinked subtasks. Incorrectly entered passwords functioned as low-stakes failure points, prompting students to revisit prior reasoning and identify errors. In this way, even “getting it wrong” offered opportunities for reflective observation, which align with research advocating for providing a psychologically safe learning environment [42].

The content of the scavenger hunt was mapped to varying degrees to the five steps of EBM [2], defining *what* students are expected to learn. While various EBM frameworks exist, including extended models like the seven-step approach [43], the five-step model remains the most widely adopted. These steps include *asking* a focused clinical question, *acquiring* evidence, *appraising* it critically, *applying* it and *assessing* outcomes. Tasks involving PICO construction and study design identification supported the “Ask” step. Citation-based challenges and article navigation tasks reflected the “Acquire” step, while interpreting study outcomes aligned with “Appraise. This step was also emphasized through SPSS-based analyses, allowing participants to interpret data and assess significance. Some tasks also engaged the “Apply” step by requiring participants to suggest interpretations or draw conclusions based on statistical outputs. Although the “Assess” step was not addressed in this study, future studies can incorporate narrative case studies by utilizing clinical simulations, (such as in apps like *Prognosis: Your Diagnosis* [44]). Such cases can provide a realistic, low-risk environment for learners to apply evidence-based decisions, reflect on outcomes and help close the EBM loop.

### Evaluation

Overall, participant feedback indicated moderate to high levels of satisfaction, with many students describing the activity as engaging and distinct from traditional learning formats. In addition, nearly one-third of respondents commented on how the activity enhanced their motivation and enjoyment. Students further described the scavenger hunt as a unique and exciting experience. These aspects mirror findings from previous research indicating that students are more engaged when they perceive learning as an enjoyable and self-directed process [32, 45, 46]. The scavenger hunt format, which required learners to actively solve riddles, collaborate with peers, and progress through sequential challenges, appeared to foster sustained participation and persistence. As reflected in participant comments describing repeated attempts to solve tasks, the challenge-based structure may have contributed to a sense of accomplishment and continued effort, aligning with principles of motivational learning design.

Although the hunt was generally perceived to be difficult, participants viewed this positively, as one participant remarked “*It was tricky. That was a good thing!*” and another described it as *“a good learning opportunity for beginners.”* This supports the notion that perceived difficulty is closely linked to the achievement of deeper learning outcomes [47].

Participant reflections also indicated perceived development of core research-related skills, including problem-solving, critical thinking, persistence, and collaboration. Over half of respondents referenced these competencies when describing their experience. In particular, the riddle-based tasks and password-generation mechanism were frequently cited as elements that required brainstorming, iterative problem-solving, and perseverance. This reflects the value of experiential, challenge-based learning activities in cultivating transferable research skills. By engaging learners in active task completion, reflection, and re-application of knowledge, the scavenger hunt operationalized key elements of experiential learning theory.

### Virtual Scavenger Hunt as Alternative Assessment

In addition to being a teaching strategy, the scavenger hunt functioned as a formative assessment that highlighted practical skill gaps. Analysis of task performance revealed that participants performed well on items assessing theoretical knowledge or recognition-based competencies. As seen in Table 1, Tasks 1 and 6a that focused on identifying appropriate statistical tests had high accuracy rates of 95% and 90%, respectively. Similarly, tasks involving the extraction of straightforward information (e.g., bibliographic details and study design) also showed strong performance, with Task 3b and Task 5c scoring 90% accuracy.

However, accuracy declined significantly in tasks that required consulting and interpreting actual research articles. For instance, only 30% of pairs correctly formulated a search string (Task 2b), and just 25% correctly identified the values of reported outcomes from a real article (Task 6c). The activity, thus, exposed a discrepancy between theoretical understanding and applied competence, therefore serving both as a diagnostic tool and a pedagogical strategy. This reaffirms literature advocating for alternative assessment methods that promote autonomy and engagement [31]. Participant feedback echoed this value, describing the experience as “*an open-book quiz*” and “*a thrilling way to educate*,” reinforcing the idea that assessment can be both diagnostic and intrinsically motivating.

### Implementation Feasibility

Beyond learner perceptions and task performance, this study provides insight into the practical feasibility of implementing a game-based approach to EBM education. The intervention was delivered within a defined, short-term timeframe using widely available digital tools, minimizing the need for specialized infrastructure or sustained faculty oversight. Facilitation was primarily limited to real-time clarification and support, suggesting that similar activities could be integrated into existing curricula without substantial additional staffing demands. The virtual delivery format further enabled participation across institutions and academic levels, supporting scalability and flexibility in scheduling and access. These features are particularly relevant for educational settings with limited physical resources or distributed learner populations.

Although this study operationalized the SEARCH framework through a virtual scavenger hunt, its design principles are transferable to other game-based and experiential formats. For example, riddle-based progression may be adapted into time-bound challenges in escape rooms, scenario-driven decision points in simulations, or role-based tasks in live-action role play (LARP) activities, while preserving the same underlying pedagogical structure.

Moreover, although a virtual format was selected in this context to enhance flexibility and scalability, the framework is equally applicable to in-person or blended implementations, depending on local resources and pedagogical goals.

## Limitations and Future Directions

Several limitations of this study should be acknowledged. The intervention was implemented in a single iteration with a relatively small participant group, which may limit generalizability. In addition, recruitment was voluntary, introducing the potential for self-selection bias, as students with a pre-existing interest in research or game-based learning may have been more likely to participate. The study did not formally assess behavioral change or long-term skill transfer, and therefore conclusions are limited to feasibility, task performance and perceived educational value.

Moreover, remote delivery inherently limits the ability to fully verify individual engagement or prevent external assistance, which could have influenced task performance. The scavenger hunt was also perceived as difficult by many participants, suggesting that future implementations may benefit from a more graduated increase in task complexity or additional scaffolding to support learners with varying levels of prior research experience. Data collection relied primarily on self-reported feedback, which, while useful for gauging perceptions, may not fully capture depth of learning or objective skill acquisition.

Despite these limitations, these findings contribute to the growing body of research advocating for interactive and student-centered learning approaches in EBM. Traditional lecture-based methods, while valuable, may not fully cultivate the higher-order thinking skills required for effective evidence-based practice. Future studies could employ pre- and post-intervention assessments to provide further insight into the effectiveness of scavenger hunts as a pedagogical tool. Moreover, studies exploring the longitudinal impact of GBL interventions on research competency should be conducted. Comparative studies evaluating scavenger hunts against traditional instructional formats would also help clarify the relative efficacy of this approach. Moreover, researchers can implement narrative case studies to evaluate the Assess step of the EBM cycle.

## Conclusion

This study demonstrates that a virtual scavenger hunt can serve as a theory-driven and feasible approach to supporting the development of research competencies in medical education. Anchored in Self-Determination Theory and Experiential Learning Theory and aligned with the steps of EBM, the activity promoted engagement, collaboration, and active application of research concepts.

Beyond reporting an educational activity, this work addresses a recognized gap in health professions education regarding how GBL activities can be designed and implemented. The SEARCH framework provides a model that offers practical guidance for educators seeking to integrate GBL into EBM curricula. Importantly, this study demonstrates task designs that require interpretation, contextual judgment and applied reasoning, thereby prioritizing learning processes that are not easily automated by AI tools. The low-resource and scalable nature of the reported activity supports its adaptability across diverse educational contexts. It contributes a replicable method for cultivating research literacy and EBM fluency in an engaging and student-centered way, with implications for broader curricular innovation in evidence-based education.

## Appendix

**Table 2:** Learning objectives and concepts related to virtual scavenger hunt tasks

Task No.	Sample “clues”/ Tasks	Related learning objectives	EBM Step
1a	Identify the test described in the riddle.	Learn to distinguish between ANOVA and other statistical methods (e.g., t-test) based on given data.Understand the foundational assumptions of ANOVA (e.g Normality, Homogeneity of Variances).	Appraise
1b	Identify from the given table where ANOVA has been used.	Compare the use cases for an Independent Sample t-test vs. ANOVA.	
2a	Build a PICO Table for the given topic:“Efficacy and safety of patiromer for hyperkalemia: A Systematic Review and Meta-Analysis”	Learn to structure clinical questions using the PICO framework.Develop skills in constructing effective search strings for systematic literature searches (Boolean Operators)	Ask, Acquire
2b	Identify the MeSH term and construct a search string for the given topic. Report the number of studies retrieved.	Understand how to execute searches and interpret results in medical databases such as PubMed (Database Search Techniques)..Be able to identify MesH terms.	
3a	Identify the citation style used in the given citation.	Familiarization with different citation styles and structures.	Acquire
3b	Extract volume number from the citation.	Identify key components of a bibliographic citation and accurately extract relevant information from them.	
4	Identify the graph in the riddle.Find the figure corresponding to the specific graph in the article attached.	Identify different types of data visualization techniques.	Appraise
5a	Identify the study design in the riddle. (Refer to Figure 2 for example task).	Recognize and define key study designs.	Appraise
5b	Find the first reference that corresponds to the study design in the riddle in the article attached.	Identify study designs used in published research articles based on methodology and language cues.	
5c	Identify the study design used in the article attached.	Familiarize participants with the structure of research articles and how to identify key study elements such as study design and supporting references.	
6a	Identify the test in the riddle.	Recognize when logistic regression is used and what types of outcomes it models.	Appraise
6b	Identify the reported outcomes of this test in the attached article.	Interpret odds ratios as reported in medical research.	
6c	Give the highest value of the reported outcome in the given article	Develop familiarity with statistical reporting in health research articles.	
7a	SPSS Based Task:From the given dataset, compare average “Sleepiness” scores between smokers and non-smokers using appropriate test in SPSS. Interpret findings and report p-value.	Learn how to apply statistical tests and interpret statistical outputs.	Appraise,Apply
7b	Subtask: Based on the result, would you consider screening smokers more often for sleep disorders?	Apply evidence to make screening recommendations.	
